# Supplementary material for: Impact of Socioeconomic Deprivation on the Local Spread of COVID-19 Cases Mediated by the Effect of Seasons and Restrictive Public Health Measures: A Retrospective Observational Study in Apulia Region, Italy
Source: Int J Environ Res Public Health. 2022 Sep 10;19(18):11410. doi: 10.3390/ijerph191811410 (PMC9517341; doi:10.3390/ijerph191811410)
Supplement: Supplementary file 1 [file ijerph-19-11410-s001.zip › ijerph-1878770-supplementary.pdf]

## Supplementary material

**Table S1.** Percentiles of the Deprivation Index distribution for heat maps in Figure 1.

| Percentile | Deprivation Index |
|---|---|
| 5th | -2.23 |
| 10th | -1.88 |
| 15th | -1.69 |
| 20th | -1.51 |
| 25th | -1.33 |
| 30th | -1.26 |
| 35th | -1.14 |
| 40th | -1.01 |
| 45th | -0.90 |
| 50th | -0.78 |
| 55th | -0.68 |
| 60th | -0.59 |
| 65th | -0.49 |
| 70th | -0.30 |
| 75th | -0.20 |
| 80th | -0.02 |
| 85th | 0.13 |
| 90th | 0.38 |
| 95th | 0.89 |

**Table S2.** Percentiles of the Incidence Rates (rates for 100,000 inhab.) distribution for heat maps in Figure 1.

| Period | | Percentile | | | | | | |
|---|---|---|---|---|---|---|---|---|
| | | 5th | 10th | 25th | 50th | 75th | 90th | 95th |
| Entire period | | 0.0 | 0.0 | 14.9 | 41.9 | 118.4 | 206.7 | 270.7 |
| 2020 | March | 0.0 | 0.0 | 3.7 | 12.9 | 20.0 | 30.5 | 43.0 |
| | April | 0.0 | 6.8 | 11.7 | 17.2 | 25.6 | 39.0 | 72.5 |
| | May | 0.0 | 0.0 | 0.0 | 7.0 | 12.8 | 19.1 | 27.1 |
| | June | 0.0 | 0.0 | 0.0 | 0.0 | 3.0 | 6.2 | 6.9 |
| | July | 0.0 | 0.0 | 0.0 | 0.0 | 2.6 | 17.8 | 29.1 |
| | August | 0.0 | 0.0 | 4.6 | 10.1 | 20.4 | 42.9 | 65.5 |
| | September | 0.0 | 0.0 | 9.0 | 16.0 | 23.1 | 33.4 | 49.1 |
| | October | 11.0 | 16.8 | 28.3 | 45.1 | 76.9 | 107.6 | 135.8 |
| | November | 81.5 | 104.8 | 125.2 | 149.1 | 233.0 | 283.6 | 339.0 |
| | December | 66.1 | 74.8 | 97.1 | 122.4 | 186.3 | 267.2 | 288.3 |
| 2021 | January | 75.5 | 88.0 | 114.1 | 143.8 | 178.4 | 226.8 | 270.0 |
| | February | 55.2 | 65.9 | 88.0 | 127.2 | 170.7 | 197.0 | 220.3 |
| | March | 105.7 | 126.0 | 175.5 | 226.7 | 272.8 | 329.0 | 413.7 |
| | April | 118.7 | 130.8 | 156.5 | 202.9 | 278.9 | 315.4 | 346.5 |
| | May | 35.4 | 39.9 | 61.8 | 89.2 | 118.5 | 151.2 | 168.1 |
| | June | 3.9 | 11.9 | 17.2 | 23.6 | 32.4 | 42.3 | 62.8 |
| | July | 4.5 | 7.4 | 11.1 | 24.3 | 40.0 | 53.4 | 72.9 |
| | August | 25.7 | 33.5 | 38.1 | 47.7 | 65.7 | 86.1 | 91.5 |
| | September | 17.2 | 21.1 | 26.1 | 38.0 | 51.7 | 71.6 | 90.2 |
| | October | 12.2 | 15.0 | 20.5 | 29.3 | 52.7 | 87.2 | 99.3 |
| | November | 23.9 | 29.0 | 34.5 | 47.2 | 57.8 | 75.7 | 93.4 |
| | December | 42.3 | 43.8 | 63.0 | 97.9 | 210.7 | 777.4 | 839.6 |

**Table S3.** Estimate Rate Ratio (RR) between age classes.

| **Comparison between age classes** | **RR [CI 95%]** |
|---|---|
| 0-5 vs 6-14 | 0.51 [0.46-0.55] |
| 0-5 vs 15-25 | 0.53 [0.45-0.63] |
| 0-5 vs 26-45 | 0.23 [0.19-0.27] |
| 0-5 vs 46-65 | 0.20 [0.17-0.25] |
| 0-5 vs 66-75 | 0.46 [0.40-0.53] |
| 0-5 vs 76+ | 0.49 [0.43-0.57] |
| 6-14 vs 15-25 | 1.06 [0.95-1.17] |
| 6-14 vs 26-45 | 0.45 [0.40-0.50] |
| 6-14 vs 46-65 | 0.40 [0.35-0.46] |
| 6-14 vs 66-75 | 0.91 [0.84-0.99] |
| 6-14 vs 76+ | 0.98 [0.90-1.07] |
| 15-25 vs 26-45 | 0.43 [0.40-0.45] |
| 15-25 vs 46-65 | 0.38 [0.36-0.41] |
| 15-25 vs 66-75 | 0.86 [0.82-0.91] |
| 15-25 vs 76+ | 0.93 [0.86-0.99] |
| 26-45 vs 46-65 | 0.90 [0.87-0.93] |
| 26-45 vs 66-75 | 2.02 [1.90-2.15] |
| 26-45 vs 76+ | 2.17 [2.01-2.34] |
| 46-65 vs 66-75 | 2.25 [2.08-2.44] |
| 46-65 vs 76+ | 2.42 [2.22-2.64] |
| 66-75 vs 76+ | 1.07 [1.03-1.12] |

**Table S4.** Estimate Rate Ratio [95%CI*] of the pairwise comparison between Deprivation Index, by and Season.

| Pairwise comparison | Season | | | |
|---|---|---|---|---|
| | Autumn | Winter | Spring | Summer |
| VH vs H | **1.66 [1.07-2.57]** | 1.37 [0.99-1.91] | 1.29 [0.90-1.86] | 1.39 [0.75-2.57] |
| VH vs M | **2.07 [1.17-3.67]** | **1.76 [1.19-2.61]** | 1.35 [0.90-2.02] | 1.41 [0.69-2.88] |
| VH vs L | **3.51 [2.25-5.48]** | **2.61 [1.81-3.78]** | **1.73 [1.16-2.6]** | 1.33 [0.71-2.50] |
| VH vs VL | **3.83 [1.88-7.81]** | **3.33 [1.86-5.97]** | **2.27 [1.10-4.65]** | 1.40 [0.65-3.02] |
| H vs M | 1.25 [0.68-2.28] | 1.28 [0.88-1.87] | 1.04 [0.66-1.64] | 1.01 [0.55-1.87] |
| H vs L | **2.12 [1.31-3.43]** | **1.90 [1.34-2.70]** | 1.34 [0.85-2.10] | 0.96 [0.58-1.58] |
| H vs VL | **2.31 [1.11-4.82]** | **2.43 [1.37-4.29]** | 1.75 [0.83-3.70] | 1.01 [0.52-1.97] |
| M vs L | 1.69 [0.92-3.11] | 1.48 [0.98-2.24] | 1.29 [0.79-2.09] | 0.95 [0.51-1.76] |
| M vs VL | 1.85 [0.81-4.21] | **1.89 [1.03-3.48]** | 1.68 [0.78-3.63] | 1.00 [0.47-2.14] |
| L vs VL | 1.09 [0.52-2.29] | 1.28 [0.71-2.31] | 1.31 [0.61-2.82] | 1.05 [0.53-2.07] |

DI, Deprivation Index; VH, Very High DI; H, High Di; M, Medium DI; L, Low DI; VL, Very Low DI; RR, Rate Ratio; CI, Confidence Interval.

*adjusted by Tukey.

**Table S5.** Estimate Rate Ratio [95%CI*] of the pairwise comparison between Deprivation Index, by and Phase.

| Pairwise comparison | Level of restrictions | | | |
|---|---|---|---|---|
| | Ph1 | Ph2 | Ph3 | Ph4 |
| VH vs H | 1.46 [0.56-3.86] | **1.57 [1.18-2.09]** | 1.54 [0.96-2.47] | 1.16 [0.85-1.58] |
| VH vs M | 1.55 [0.46-5.17] | **2.05 [1.51-2.78]** | **1.87 [1.24-2.81]** | 1.17 [0.82-1.66] |
| VH vs L | 2.29 [0.82-6.34] | **2.78 [2.06-3.75]** | **2.39 [1.50-3.82]** | **1.40 [1.04-1.88]** |
| VH vs VL | 1.62 [0.2-13.26] | **3.36 [2.35-4.82]** | **3.63 [1.80-7.31]** | **2.06 [1.53-2.77]** |
| H vs M | 1.06 [0.31-3.60] | 1.31 [0.95-1.79] | 1.21 [0.73-2.02] | 1.01 [0.72-1.42] |
| H vs L | 1.56 [0.55-4.43] | **1.77 [1.30-2.41]** | 1.55 [0.89-2.71] | 1.21 [0.91-1.60] |
| H vs VL | 1.11 [0.13-9.16] | **2.14 [1.48-3.09]** | **2.36 [1.10-5.05]** | **1.78 [1.34-2.36]** |
| M vs L | 1.48 [0.42-5.24] | 1.36 [0.98-1.88] | 1.28 [0.77-2.12] | 1.20 [0.86-1.66] |
| M vs VL | 1.05 [0.11-9.75] | **1.64 [1.12-2.40]** | 1.94 [0.94-4.01] | **1.76 [1.27-2.45]** |
| L vs VL | 0.71 [0.08-6.01] | 1.21 [0.83-1.76] | 1.52 [0.71-3.24] | **1.47 [1.13-1.92]** |

DI, Deprivation Index; VH, Very High DI; H, High Di; M, Medium DI; L, Low DI; VL, Very Low DI; RR, Rate Ratio; CI, Confidence Interval; Ph1, total lockdown; Ph2, soft lockdown; Ph3, moderate restrictions; Ph4, low restrictions.

*adjusted by Tukey.
